# Presumptive Development of Fibrotic Lung Disease From *Bordetella bronchiseptica* and Post-infectious Bronchiolitis Obliterans in a Dog

**DOI:** 10.3389/fvets.2019.00352

**Published:** 2019-10-10

**Authors:** Jared A. Jaffey, Mark Harmon, Isabelle Masseau, Kurt J. Williams, Carol Reinero

**Affiliations:** ^1^Department of Veterinary Medicine and Surgery, Veterinary Health Center, University of Missouri, Columbia, MO, United States; ^2^Department of Clinical Sciences, Université de Montréal, Saint-Hyacinthe, QC, Canada; ^3^Department of Pathobiology and Diagnostic Investigation, College of Veterinary Medicine, Michigan State University, East Lansing, MI, United States

**Keywords:** pulmonary fibrosis, canine infectious respiratory disease complex (CIRDC), pulmonary hypertension, pneumonia, interstitial lung disease, PIBO

## Abstract

A 7-month-old Miniature Poodle acquired from a pet store developed cough and subsequently respiratory distress compatible with *Bordetella bronchiseptica* infection. Partial but incomplete resolution of clinical signs and thoracic radiographic/computed tomographic imaging lesions were noted with use of susceptibility-guided antimicrobials. Additionally, a concern for an infectious nidus led to left cranial lung lobectomy at 9 months of age. Histopathology predominantly revealed polypoid and constrictive bronchiolitis obliterans (i.e., small airway disease). Intermittent antimicrobial administration over the next 5 years failed to blunt progressive clinical signs. At 8 years, necropsy confirmed severe airway-centered interstitial fibrosis. This pattern of fibrosis was strongly suggestive of underlying small airway disease as the trigger. In retrospect, post-infectious bronchiolitis obliterans (PIBO), a syndrome in young children caused by pulmonary infections but not yet recognized in pet dogs, likely initiated a pathway of fibrosis in this dog. In dogs with risk factors for community-acquired pathogens such as *Bordetella bronchiseptica*, PIBO is a differential diagnosis with development of severe, persistent respiratory signs incompletely responsive to appropriate antimicrobials. Untreated PIBO may lead to airway-centered interstitial fibrosis. Future study is required to determine if targeted therapy of PIBO could alter the course of end-stage pulmonary fibrosis.

## Background

Post-infectious bronchiolitis obliterans (PIBO), a syndrome in children most commonly caused by *Mycoplasma pneumonia* (1, 2) and adenovirus (1) and occasionally *Bordetella pertussis* (3, 4) is associated with chronic inflammatory and fibrotic lesions of small airways leading to chronic airflow obstruction (5). Treatment is generally supportive therapy and frequently includes glucocorticoids (4–6). In dogs, while many contagious respiratory pathogens cause tracheobronchitis and pneumonia, bronchiolar diseases including PIBO are not well recognized as spontaneous clinical syndromes. Importantly, severe damage to the lung can lead to end-stage and untreatable fibrosis, with most cases in dogs not having a recognizable trigger and thus being termed “idiopathic pulmonary fibrosis.” This report describes a puppy developing PIBO after *Bordetella bronchiseptica* pneumonia with histologic evidence of small airway changes strongly supporting development of pulmonary fibrosis. Recognizing addressable triggers of fibrotic lung disease could have important implications for delaying progression of end-stage lung lesions.

## Case Presentation

A 7-month old male intact Miniature Poodle presented to the University of Missouri Veterinary Health Center with acute respiratory distress. Previously the primary care veterinarian treated it for cough with successive 2-week courses of amoxicillin/clavulanic acid, doxycycline, and enrofloxacin with no improvement. It had been purchased 2 months prior from a pet store and was not current on vaccines or parasiticides and canine infectious respiratory disease complex (CIRDC) was a top differential. Upon presentation, the owner reported the puppy had increased respiratory effort, tachypnea, weakness, and hacking cough for 2 days. Physical examination findings included heart rate of 120 beats/min, respiratory rate of 60 breaths/min, and pyrexia 103.8°F (39.9°C). Cardiothoracic auscultation revealed crackles in all lung fields, with absence of murmur or arrhythmia. A blind bronchoalveolar lavage (BAL) showed numerous intra- and extracellular bacteria consistent with septic suppurative pneumonia with light growth of *Bordetella bronchiseptica* on enrichment broth. The reason for the pure growth of *Bordetella bronchiseptica* only on enrichment broth could not be determined by the historic medical record; possible explanations could have been a recent unrecorded dose of an antimicrobial or improper sample handling prior to submission. A respiratory polymerase chain reaction assay on an endotracheal tube swab was negative for *Bordetella bronchiseptica*, Influenza A virus, adenovirus-2, distemper, herpesvirus, parainfluenza, respiratory coronavirus, and *Streptococcus equi* subsp. *zooepidemicus*. In light of these results and sensitivity testing, the dog was treated with sulfamethoxazole/trimethoprim (12.5 mg/kg PO q12 h); additionally, fenbendazole (23.4 mg/kg PO q24 h) for 5 days was administered.

Ten days later the dog re-presented with respiratory decompensation despite antimicrobial therapy guided by susceptibility testing. Physical examination findings included heart rate of 100 beats/min, respiratory rate of 60 breaths/min, temperature of 100.8°F (38.2°C), 5% dehydration, and cyanotic oral mucous membranes. Cervical tracheal palpation elicited a hacking cough. Cardiothoracic auscultation revealed crackles and soft wheezes in all lung fields. Pulse oximetry yielded a hemoglobin saturation of 84%, (reference range, 95–100%) while breathing room air. Supplemental oxygen via intranasal prongs was provided.

**Figure 1:**
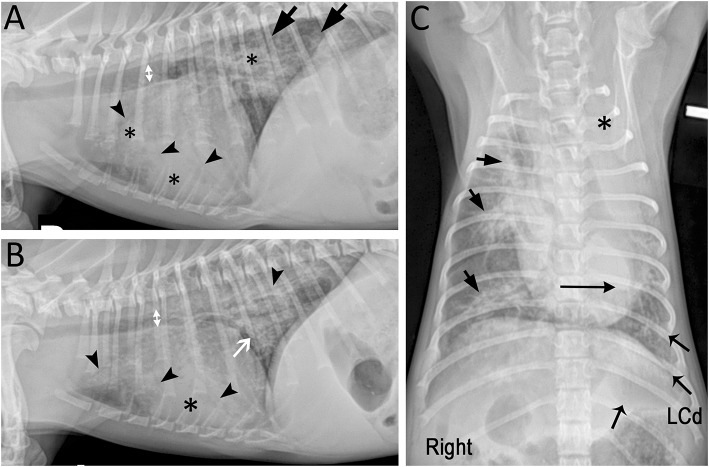
Marked mixed pulmonary pattern with diffuse radiographic lesions in a 7-month-old Miniature Poodle presented with respiratory decompensation while receiving appropriate antimicrobials. **(A)** Right and **(B)** left lateral radiographic projections showing bilateral diffuse lesions characterized by marked diffuse small airway thickening (white arrow), peribronchial cuffing and multifocal alveolar pattern (*) with air bronchograms (arrowheads). Incidentally, intra-thoracic tracheal luminal diameter (doubleheaded arrows) varied on average 28% between both lateral projections suggesting dynamic tracheal collapse (7). The tracheal bifurcation and principal bronchus were narrowed on the left lateral projection. **(C)** On the ventrodorsal projection, the left cranial lung lobe is completely opacified (*) and the cardiac silhouette is shifted to the left (long horizontal arrow). The lesions are centered around the lobar and segmental bronchi (short arrows) and decrease in severity toward the periphery. The left caudal lung lobe (LCd; flared arrows) is the second most severely affected lobe after the left cranial lobe.

Thoracic radiographs indicated diffuse changes consistent with pneumonia including consolidation of multiple lung lobes and atelectasis of the left cranial lobe (Figure 1). Repeat BAL revealed predominantly degenerate neutrophils and numerous extracellular/intracellular bacteria. Culture of BAL showed heavy growth of only *Bordetella bronchiseptica*. Enrofloxacin (10 mg/kg IV q24 h) was administered based on antimicrobial susceptibility. Hypoxemia and pyrexia resolved but tachypnea and cough persisted. The dog was discharged after 8 days of hospitalization. Treatment included orbifloxacin (6 mg/kg PO q24 h) for 4 weeks, sterile saline nebulization and coupage every 12 h for 2 weeks. The dog showed improvement but not resolution of cough, respiratory rate/effort, and energy during 4 weeks of treatment. After discontinuation of orbifloxacin clinical signs worsened. Orbifloxacin administered for 2 more weeks did not lead to improvement.

**Figure 2:**
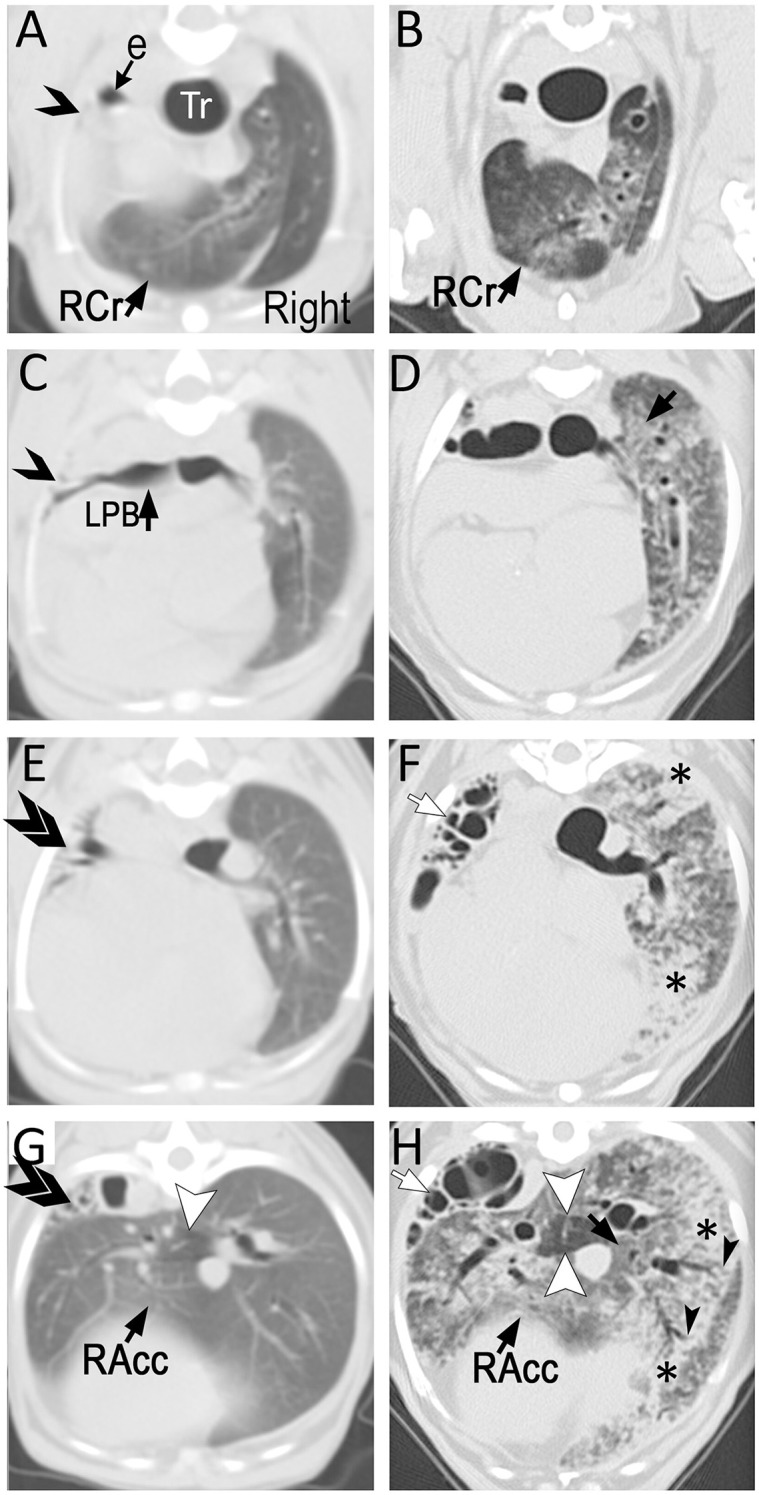
Transverse (3 mm-thick) computed tomographic (CT) images of a Miniature Poodle obtained at 9 months (**A,C,E,G**; using a single slice CT) and 8 years (**B,D,F,H**; using a 64-detector row CT) of age displayed from the same level side by side. On the first study, the left cranial lung lobe (single opened arrow) is completely collapsed with almost complete absence of air in the airways. The caudal branch of the left cranial lobar bronchus is narrowed but filled with air (ventral to opened arrow on **C**). The left caudal lung lobe is also severely reduced in volume and thus, increased in attenuation with several air-filled end on airways (double arrows). The right lung is hyperinflated with the right cranial (RCr) and accessory (RAcc) lobes extending to the left of midline. Mosaic pattern characterized by a well circumscribed hypoattenuating area is seen in the accessory lobe (arrowhead). The parenchyma is otherwise relatively normal. By 8 years of age and after undergoing left cranial lobectomy as a puppy, the entire hyperinflated right lung has developed severe patchy parenchymal lesions consisting of ground-glass opacification (black arrows) and consolidation (*). This latter is most severe surrounding medium- to large-caliber dilated airways. Traction bronchiectasis (black arrowheads) in areas of architectural distortion is seen at the periphery of several of these airways, a common feature seen with pulmonary fibrosis. The mosaic pattern previously recognized in the accessory lobe (white arrowheads) is accentuated. Several cystic air-filled structures (white arrows) of varying size occupy the left caudal lobe.

Thoracic computed tomography (CT, single slice CT scanner, Picker PQ 6000, Phillips Medical Systems, North America, Bothell, WA, USA) was performed to investigate an underlying cause for chronic cough and intermittent labored respiration (Figures 2A,C,E,G). Severe reduction in left lung volume was compensated by hyperinflation of the right lung, leading to medial displacement of right cranial and accessory lung lobes. Several end on gas-filled structures were seen in left lung lobes. Tracheal bifurcation and left principal bronchus were slightly collapsed. No additional diagnostics were performed to look for evidence of an underlying immune defect.

**Figure 3:**
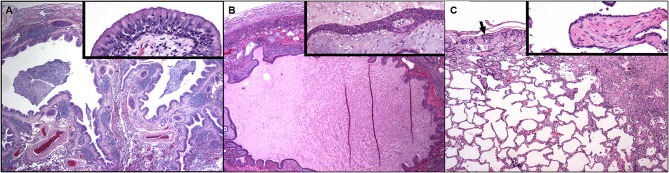
Dog lung at 9 months of age **(A)** and again at the time of post-mortem examination 7 years later **(B,C)**. **(A)** Two bronchial profiles are dilated and contain mucus and inflammatory cells within their lumens and the mucosa is thickened. There is compression and collapse of the surrounding alveolar parenchyma. Pulmonary arterial walls are markedly thickened. Hematoxylin and eosin. Inset: High magnification of the airway mucosa. The luminal epithelium is hyperplastic, protrudes into the lumen and is underlain by thickened lamina propria. Numerous neutrophils are present within the hyperplastic epithelium. (Hematoxylin and eosin.) **(B)** Dog lung at the time of post-mortem examination. A markedly dilated airway contains eosinophilic proteinaceous fluid and cellular debris. There is compression and collapse of the surrounding alveolar parenchyma. The mucosa is irregular and the lamina propria contains myxomatous material and chronic inflammation. Inset: High magnification of the airway mucosa. There is squamous metaplasia of the mucosal epithelium and abundant myxomatous matrix is within the lamina propria. (Hematoxylin and eosin.) **(C)** Away from the dilated airways regions of subpleural fibrosis with metaplastic epithelium (arrow) as well as fibrosis in the deeper parenchyma is present. Inset: A well-organized plug of fibrous connective tissue (polypoid bronchiolitis obliterans) extends into the lumen of a respiratory bronchiole.

Left-sided thoracotomy and left cranial lung lobectomy was performed because of failure of multiple antimicrobials, and in case this lobe was a nidus for infection. Histopathology of the left cranial lung lobe demonstrated small airway disease consisting of intraluminal plugging of bronchioles with downstream honeycombing, air trapping and subpleural fibrosis (Figure 3A); the upstream bronchiolar lesions strongly supported small airways disease as the cause of the fibrosis. Larger airways (bronchi) were similarly narrowed with epithelial and goblet cell hyperplasia, intraluminal mucus and mucosal and submucosal inflammatory cell infiltration. Special stains failed to identify bacteria/microbes. Interestingly, culture of lung tissue showed heavy growth of *Bordetella bronchiseptica*. The absence of histologic evidence of infectious bronchopneumonia or positively staining bacterial organisms would strongly argue against *Bordetella* as a pathogen at the time of biopsy. However, as small airway disease was not recognized as a clinical syndrome in dogs at that time, the positive culture of *Bordetella bronchiseptica* was treated with marbofloxacin (5.2 mg/kg PO q24 h) for 28 days in accordance with antimicrobial susceptibility.

**Figure 4:**
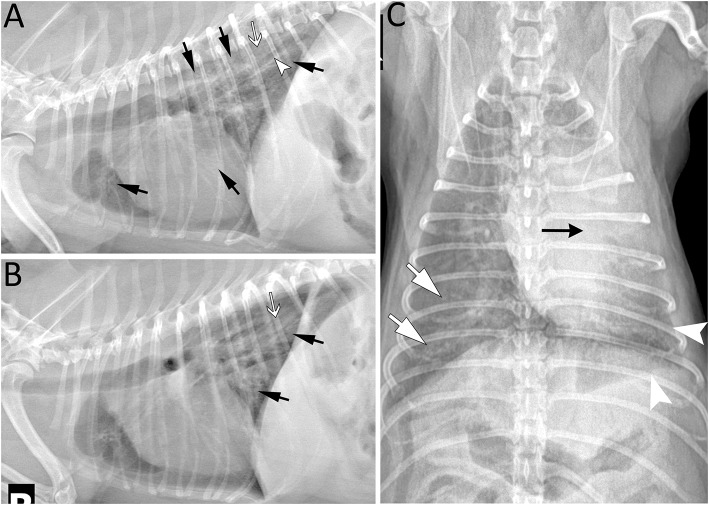
Five years post left cranial lobectomy, radiographic lesions were still present especially in the left caudal lung lobe of a 6-year-old Miniature poodle. **(A)** Left lateral projection showing several areas of increased opacity in the cranioventral, caudoventral and caudodorsal lung field (arrows). In the caudodorsal lung field, a large bronchus (arrowhead) is dilated and does not taper as it extends toward the periphery indicating bronchiectasis. In the more severe opacified areas, small air bronchograms are seen (white arrow). **(B)** Right lateral projection revealing radiographic lesions (black and white arrows) similar to those on the left and most prominent in the caudodorsal lung field. They are less extensive in the ventral aspect of the thorax in comparison to the left lateral view. Note that some lesions maybe less distinct due to motion artifact. **(C)** As expected following left cranial lobectomy, leftward mediastinal shift remains visible (black arrow) on the ventrodorsal projection. The borders of the left caudal lobe appear retracted (arrowhead) and the lung lobe is increased in opacity. Lesions are most severe centrally and less extensive toward the periphery. Similarly, radiographic opacity is increased (white arrows) surrounding major lobar structures (vessels and bronchus) of the right lung and gradually diminish in periphery of the lung.

The dog was managed solely by his primary care veterinarian with courses of antimicrobials over the next 5 years; complete resolution of respiratory signs had never been achieved. Cough and eventually exercise intolerance were progressive. At 6 years of age, it returned to the University of Missouri Veterinary Health Center for evaluation of acute hemorrhagic diarrhea syndrome. Inspiratory crackles were noted on examination. Arterial blood gas analysis revealed hypoxemia (P_a_O_2_ = 41 mmHg; F_I_O_2_ = 0.21). Thoracic radiographs revealed an alveolar pattern and air bronchograms, bronchial thickening and unstructured interstitial pattern caudodorsally and occasionally in the dependent lung field (Figure 4). Echocardiography revealed moderate pulmonary hypertension without left-sided cardiac disease (Supplementary Figure 1). Continuous-wave Doppler assessment of peak tricuspid regurgitation was 3.49 m/s resulting in a tricuspid regurgitation pressure gradient between the right atrium and right ventricle of 48.7 mmHg (calculated using the modified Bernoulli equation, Δp = 4V^2^). Thoracic CT with angiography, bronchoscopy, and BAL were recommended but declined. In lieu of a definitive diagnosis the dog was treated with sildenafil (1.3 mg/kg PO q12 h) and enrofloxacin (5.8 mg/kg PO q 12 h) for pulmonary hypertension and possible bronchopneumonia, respectively. The dog clinically improved and was discharged 4 days later. The dog was treated with sildenafil indefinitely.

Approximately 2 years later the dog returned for evaluation of respiratory distress. In the intervening 2 years the dog's cough and exercise intolerance worsened substantially. Pertinent physical examination findings included heart and respiratory rates of 180 beats and 98 breaths per min, cyanotic mucous membranes and diffuse inspiratory crackles. The dog was treated with 40% inhalational oxygen, furosemide (2 mg/kg IV once), pimobendan (0.32 mg/kg PO once), and butorphanol (0.1 mg/kg IV once).

The following day, the dog was breathing at 108 breaths/min in an orthopneic posture. Because of progressively worsening respiratory distress and grave prognosis, euthanasia was elected. Immediately before euthanasia (performed while the dog was under general anesthesia), thoracic CT (64-detector row Toshiba Aquilion, Toshiba America Medical Systems, Tustin, CA) was performed with owner consent. The dog was intubated and mechanically ventilated (Engstrom Carestation ventilator, GE Healthcare). Ventilator settings included inspired oxygen concentration of 40%, tidal volume 10 mls/kg, respiratory rate 10 breaths/min, and positive end-expiratory pressure (PEEP) 5 cm H_2_O. Inspiratory and expiratory breath holds were ventilator-assisted and performed in tandem with CT scans; PEEP was set to 0 cm H_2_O for the expiratory breath hold. Contiguous 2 mm transverse images were obtained from the level of C6 to L2 with the dog in sternal recumbency. The left caudal parenchyma was absent and replaced by small to large air-filled structures representing honeycombing. In contrast to the prior CT, the right lung now demonstrated patchy ground-glass opacities to consolidation. The latter was mostly distributed surrounding large to medium caliber airways, the majority of which were dilated, and accompanied by architectural distortion and traction bronchiectasis (Figures 2B,D,F,H).

Following euthanasia, lungs were removed with owner consent and fixed for histopathology. To prevent collapse and disruption of lung structures, each lobe was inflation-fixed with 10% formalin. Histopathologic examination of left cranial, right caudal and accessory lung lobes was performed. In left cranial and right caudal lung lobes there was marked multifocal chronic polypoid and constrictive bronchiolitis obliterans with multifocal alveolar interstitial fibrosis (Figure 3B). This pattern of fibrosis radiating from and encompassing the diseased small airways is called airway-centered interstitial fibrosis and highlights bronchiolar pathology as the contributor to fibrosis (Figure 3C). Pulmonary arterial medial hypertrophy and intimal hyperplasia were noted. The accessory lung lobe had severe bronchiolar ectasia with collapse of surrounding alveolar parenchyma and severe neutrophilic and macrophagic inflammation. There was no evidence of infection including with special stains.

## Discussion

Sustained damage to terminal and respiratory bronchioles postulated secondary to *Bordetella bronchiseptica* possibly in combination with additional unidentified CIRDC pathogens (e.g., adenovirus type 2 and/or *Mycoplasma* spp.) early in life, led to biopsy-confirmed polyploid and constrictive bronchiolitis obliterans in an 9 month-old puppy. Post-infectious bronchiolitis obliterans, a syndrome in children that can be caused by adenovirus, *Mycoplasma pneumonia*, and less commonly *Bordetella pertussis*, is treated after resolution of infection with supportive therapies and corticosteroids to target inflammation (1–3). As PIBO is not a recognized syndrome in dogs, this dog failed to receive corticosteroid therapy, likely culminating in end-stage pulmonary fibrosis inclusive and distal to affected airways. Airway-centered interstitial fibrosis in people is one of three distinct patterns of lung fibrosis and emphasizes the site of initial injury is the small airways (8). Histologic examination as a puppy and as an 8-year-old dog showed similar lesions of bronchiolitis obliterans and downstream fibrosis, with progressive, severe and more widespread lesions (especially fibrosis) from the latter time point. With awareness of PIBO in dogs, an early trigger of pulmonary fibrosis could have potential for targeted therapy. Direct treatment of a trigger causing eventual fibrosis represents a paradigm shift from the current strategy of attempting to treat fibrosis when it is end stage.

*Bordetella bronchiseptica* is the most common bacterial pathogen in CIRDC, an umbrella term encompassing multiple bacterial and viral pathogens leading to acute respiratory tract infection (9). Most cases are self-limiting and confined to the trachea and bronchi, but when there is extension to the terminal bronchioles and alveoli, bronchopneumonia results (10). *Bordetella bronchiseptica* is the most common cause of community-acquired pneumonia in puppies (11). Although chronic respiratory disease is well documented in children having viral infections in infancy (12, 13), chronic manifestations of CIRDC are poorly described. Chronic bronchitis (14) and bronchiectasis (15, 16) may be sequela. Co-infections with CIRDC are common. One recent study identified more than 1 respiratory pathogen in approximately 50% of dogs that were *Bordetella bronchiseptica*-positive using real-time PCR on nasal and pharyngeal swab samples (17). Therefore, it is possible the dog in this report was infected with ≥1 respiratory pathogen including adenovirus type 2 and/or *Mycoplasm*a spp. While adenovirus type 2 was included in the initial respiratory PCR panel it is possible it was not identified because the sample was obtained from the endotracheal tube and not from BALF or sampling was performed after the peak shedding (7–10 days) after the initial infection (18). In addition, *Mycoplasma* spp., was not included in the respiratory pathogen PCR assay nor was a culture specific for this pathogen pursued.

The clinical syndrome bronchiolitis obliterans (BO; also known as obliterative bronchiolitis or constrictive bronchiolitis) targets small airways, defined as having internal diameters of <2 mm and lacking cartilage within their walls causing a spectrum of clinical signs (19). While somewhat confusing, it is important to understand that “bronchiolitis obliterans” can be a clinical syndrome or a histologic descriptor. The former has not been characterized in pet dogs as it has been in humans and cats (19, 20) while the latter is fairly common. In humans, bronchiolar diseases can be classified as primary or secondary: in the former, pathology is anatomically limited to small airways and in the latter, disease extends either from large airways or from lung interstitium to small airways (19). Although a similar classification scheme has been adapted to cats, distinction between primary bronchiolar disease leading to pulmonary fibrosis and interstitial lung disease culminating in fibrosis with secondary involvement of small airways may be difficult to distinguish (20). The dog in this case report with persistent and progressive clinical signs and paired CT imaging/histopathologic lung examination 7 years apart, provided support that PIBO triggered pulmonary fibrosis.

Injuries linked to BO in people include respiratory infections, chronic gastroesophageal reflux, allergic reactions, collagen vascular diseases, organ transplantation and chronic exposure to air pollutants or toxic fumes (5, 21). Experimental canine BO can be induced by respiratory infections [adenovirus (22, 23), mycoplasma (24) and co-infection of parainfluenza and *Bordetella bronchiseptica* (25, 26)], toxins (27), and allogeneic hematopoietic cell transplantation (28). Chronic small airway obstruction from inflammation and fibrosis leads to cough, wheeze and dyspnea in people (21, 29). Disease may extend to alveoli and interstitium (30). Chronic BO can lead to pulmonary fibrosis, respiratory failure, and death in people (6, 31, 32). A 1988 study showed pediatric diagnosis of BO was made post-mortem in 37% of cases (31); however, by the following decade with improved socioeconomics, increased recognition, and use of thoracic high resolution CT scans, incidence dramatically declined (4, 32). Whether a similar pattern could occur in dogs given a high index of suspicion of BO remains to be seen.

Post-infectious bronchiolitis obliterans occurs in young children after severe pulmonary infections with measles, influenza, *Bordetella pertussis, Mycoplasma pneumonia* and adenovirus (5, 31, 32). When cough and wheeze fail to resolve after acute respiratory infection, PIBO is a strong consideration (30). Supportive criteria for PIBO in lieu of lung biopsy includes history of infectious pneumonia within the first 3 years of life in otherwise healthy children; physical exam or lung function tests supporting airway obstruction; thoracic radiographic and especially thoracic CT compatible features, and exclusion of other chronic lung diseases (4, 33, 34). The dog in our report had severe *Bordetella bronchiseptica* pneumonia as a puppy, with persistent clinical signs despite appropriate antimicrobials. Histopathology confirmed BO, at the time an unrecognized clinical condition, approximately 2 months after initial diagnosis of bronchopneumonia. Honeycombing and subpleural fibrosis downstream of small airway lesions were present at the time of the initial biopsy and suggest a cause and effect relationship.

Results of thoracic radiography may be normal and are non-specific in bronchiolar diseases (5, 35). Thoracic radiography in children with PIBO classically demonstrate hyperinflation/hyperlucency, atelectasis, patchy consolidation, airway wall thickening and bronchiectasis (4, 6). As a puppy, radiographic changes reflected some changes reported in children. Thoracic CT is more sensitive than radiography and airflow limitation can be captured with paired inspiratory/expiratory scans (36, 37). Common CT findings in children with PIBO include a mosaic pattern, bronchiectasis, peribronchial thickening, atelectasis and/or consolidation (4, 36, 38). The classic finding diagnostic for air trapping is accentuation of mosaic attenuation on expiratory series which can be missed on inspiratory series alone (36, 37). The initial CT performed as a puppy occurred prior to a protocol for ventilator-acquired inspiratory and expiratory breath-holds at our facility and thus less well reflected air trapping. Prior to euthanasia, CT lesions were dominated by fibrosis although patchy regions of air trapping were noted. Histopathology of lung tissue approximately 7 years after initial lung biopsy showed marked multifocal chronic constrictive and obliterative BO with multifocal interstitial fibrosis. Therefore, it is reasonable to suspect CIRDC endured early in life contributed to chronic BO (i.e., PIBO) and eventual interstitial fibrosis as an end-stage sequela in the dog reported here. However, we cannot rule out the possibility that the dog developed BO and interstitial fibrosis for reasons not yet explored in companion animals.

Empirical therapy of PIBO in people includes glucocorticoids, bronchodilators, oxygen supplementation, mechanical ventilation or antimicrobials (4, 29). Surgical excision of bronchiectasis or segmental atelectic lung are performed if conservative therapy fails to achieve clinical improvement (4). Interestingly, our dog clinically improved after removal of the atelectic left cranial lung lobe as a puppy. The dog reported here was treated with numerous courses of various different antimicrobials by the primary care veterinarian over the course of 5 years without clinical improvement. Therapy over this time was not guided by confirmation of pathogenic organisms from airway sampling (e.g., BALF cytology, culture, or PCR). The lack of clinical improvement with multiple types and courses of antimicrobials makes repeated bacterial infections unlikely, but not impossible. Therefore, chronic-intermittent respiratory bacterial infections could not be ruled out as contributory to the development of fibrotic lung disease in this dog.

Pulmonary fibrosis (fibrotic interstitial lung disease), represents a heterogeneous group of syndromes originating from diverse injuries culminating in scar tissue and an end-stage lung (39). Fibrosis attempts to repair tissue and protect against subsequent injury by increasing tissue strength; however, repetitive injury may lead to dysregulated fibrosis that is maladaptive and detrimental (40). In dogs, fibrosis may originate secondary to genetic contributors (e.g., West Highland White terrier) or drugs/toxins, with most cases failing to have an identified trigger (39). The dog in this report strongly suggests PIBO can progress to widespread airway-centered interstitial fibrosis. It is unknown if targeted therapy (e.g., glucocorticoids) could have prevented or slowed fibrosis by diminishing inflammation leading to aberrant repair. In dogs, PIBO should be a differential diagnosis for otherwise healthy young dogs developing CIRDC pneumonia and respiratory signs persisting despite appropriate antimicrobials and with compatible thoracic imaging or histologic changes.

## Data Availability Statement

All datasets generated for this study are included in the manuscript/Supplementary File.

## Author Contributions

JJ, CR, MH, IM, and KW contributed to writing of the manuscript and literature review. KW contributed to the histopathological interpretation and description. IM and CR contributed to interpretation and description of the imaging findings. MH was responsible for interpretation of echocardiography imaging, interpretation, and description. All authors contributed to the final review of the manuscript.

### Conflict of Interest

The authors declare that the research was conducted in the absence of any commercial or financial relationships that could be construed as a potential conflict of interest.
