# Supplementary material for: Presumptive Development of Fibrotic Lung Disease From Bordetella bronchiseptica and Post-infectious Bronchiolitis Obliterans in a Dog
Source: Front Vet Sci. 2019 Oct 10;6:352. doi: 10.3389/fvets.2019.00352 (PMC6795681; doi:10.3389/fvets.2019.00352)
Supplement: Supplementary file 1 [file Data_Sheet_1.docx]

Supplementary Figure


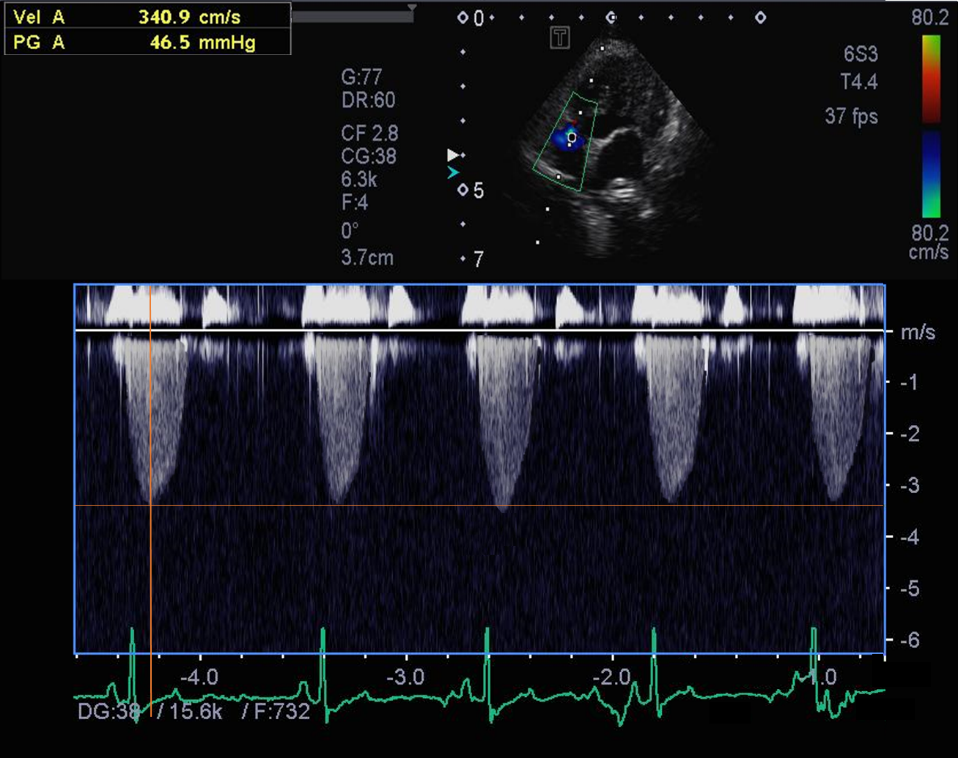


Supplementary Figure 1. Continuous wave Doppler interrogation of tricuspid regurgitation from the dog of this report. The maximal tricuspid regurgitation flow velocity is approximately 3.4 m/s, indicating a peak tricuspid gradient of approximately 46 mmHg. Assuming a normal right atrial pressure of 5 mmHg, the estimated systolic pulmonary artery pressure is approximately 51 mmHg consistent with mild-to-moderate pulmonary hypertension.
